# Spectrum Sharing Based on a Bertrand Game in Cognitive Radio Sensor Networks

**DOI:** 10.3390/s17010101

**Published:** 2017-01-07

**Authors:** Biqing Zeng, Chi Zhang, Pianpian Hu, Shengyu Wang

**Affiliations:** 1 School of Computing, South China Normal University, Guangzhou 510631, China; hu_pianpian@126.com (P.H.); wangshengyu_1992@163.com (S.W.); 2 IFLYTEK Co., Ltd., Hefei 230000, China; keepage@foxmail.com

**Keywords:** wireless sensor network, spectrum leasing, spectrum allocation, spectrum pricing, Bertrand game

## Abstract

In the study of power control and allocation based on pricing, the utility of secondary users is usually studied from the perspective of the signal to noise ratio. The study of secondary user utility from the perspective of communication demand can not only promote the secondary users to meet the maximum communication needs, but also to maximize the utilization of spectrum resources, however, research in this area is lacking, so from the viewpoint of meeting the demand of network communication, this paper designs a two stage model to solve spectrum leasing and allocation problem in cognitive radio sensor networks (CRSNs). In the first stage, the secondary base station collects the secondary network communication requirements, and rents spectrum resources from several primary base stations using the Bertrand game to model the transaction behavior of the primary base station and secondary base station. The second stage, the subcarriers and power allocation problem of secondary base stations is defined as a nonlinear programming problem to be solved based on Nash bargaining. The simulation results show that the proposed model can satisfy the communication requirements of each user in a fair and efficient way compared to other spectrum sharing schemes.

## 1. Introduction

In the field of Cyber-Physical Social Sensing, wireless sensor networks (WSNs) [1,2,3] play a very important role. WSNs are organized networks composed of some tiny sensor nodes that can perform information collection, processing and wireless transmission actions. Due to the large number of nodes in WSNs, a large amount of data needs to be collected [4,5,6], and these data also need to be transmitted, which leads to the problem of spectral tension and node limitations. In order to solve these problems, Cognitive Radio Sensor Networks (CRSNs) have become a research hotspot of many scholars at home and abroad. CRSNs make each sensor node have the ability of spectrum sensing and dynamic spectrum access by introducing cognitive radio (CR) [7,8] technology into WSNs, and is considered to be the most promising wireless sensor network of the next generation [9,10]. Dynamic spectrum sharing make cognitive user/secondary users that have CR technology share spectrum resources that can only be used by authorized user/primary users (PUs) by adaptively adjusting the transmission parameters in the CRSNs, which makes the spectrum resource utilization not be high in traditional static management mode and alleviates the shortage of spectrum resources.

According to different wireless network access methods, dynamic spectrum sharing can be divided into underlay mode [11] and overlay mode [12,13]. In underlay mode, the secondary user is able to evaluate the interference temperature threshold of the primary network, by using spread spectrum technology and control transmission power below the interference temperature threshold, then can use a very wide frequency band to obtain a higher transfer rate. In overlay mode, the secondary users detect and use idle bandwidth that is currently not occupied by the PU, namely spectrum holes [14], when the primary user comes back to the spectrum section, secondary users switch to another spectrum band through spectrum migration. The two kinds of share mode lead to two main problems: power control and spectrum allocation. In addition, during the data transmission process, the uncertainty of spectrum resources is a big problem in dynamic spectrum sharing, which has prompted the birth of another way of spectrum sharing, whereby a spectrum manager allows secondary users to get a license at a certain price, so as to ensure the certainty of the available spectrum, this way is called spectrum transaction/spectrum leasing. Spectrum pricing is another important issue in the study of dynamic spectrum sharing.

References [15,16] examined the problems of uplink transmission power allocation and power control. The base station sets the price per unit of transmission power, with the premise that every secondary user ensures its own transmission power is less than the interference temperature threshold, and the secondary user can complete up-link data transmission through paying the corresponding cost for the base station. For the problems of system spectrum pricing and allocation, references [17,18] studied the spectrum allocation problem of different market/topology structures based on the oligopoly market model. The primary user as the seller in market would determine the pricing per unit of spectrum bandwidth and lease it to the secondary users for maximum gains. The secondary users determine the quantity of purchase to maximize their own utility by the pricing and the QoS performance obtained. The game between the primary user and the secondary user could be stable in a state of balance of supply and demand in the market, namely market equilibrium. For multi-hop cognitive radio networks, [19] proposed the power control strategy based on time division multiplexing to achieve the maximum throughput from end to end. However, current studies rarely involve the spectrum pricing and allocation problem. The related research needs to ensure that the transmission power of secondary users is less than interference temperature threshold of the primary network, and the spectrum allocation of frequency and power dimension should be considered at the same time. In [20] a kind of hierarchical model to study the allocation problem of power and spectrum was proposed. However, the bandwidth demands that primary user pricing depends on is not from secondary user, and bandwidth and power had the same pricing, the power control problem was also neglected.

Furthermore, the power control model based on pricing [15,16] is mainly based on a user utility function defined by the signal to noise ratio. In spectrum research based on pricing [17,18], it was generally considered that the secondary user utility function had a positive correlation with channel capacity or spectrum efficiency, but a negative correlation with rental costs. Only part of references [20,21] considered the secondary user utility from the point of view of the secondary network demand. Reference [20] defined a utility function reflecting QoS satisfaction degree based on a sigmoid function in service providers offering terminal users power allocation. For three kinds of network scenarios including delay-sensitive secondary users, [21] studied spectrum access control based on pricing strategy. This work didn’t consider the channel capacity demand of the secondary users. By defining a utility function based on channel utility or signal to noise ratio, it is possible to make the utility value greater when the secondary base station achieves more spectrum and power resources. However, on the one hand, excess spectrum beyond the need for user communication service can be considered as a waste of resources. On the other hand, excess transmission power not only increases system interference, but also shortens the battery life of mobile users. Reference [22] studied cooperative spectrum sharing of secondary users based on the communication demands of these secondary network users. Considering both QoS needs and power constraint, [23] studied power control and spectrum to reach maximum system throughput based on mixed-integer non-linear programming. However, these works did not study the secondary user utility from the perspective of communication requirements for the spectrum price problem. The study of secondary user utility from the perspective of communication demand can not only promote the secondary users to meet the maximum communication needs, but also maximize the utilization of spectrum resources to reduce the waste of spectrum resources and excess power on the network environment caused by interference. Thus, considered the communication needs of users, this paper proposes a kind of two stage model of spectrum pricing and allocation to study the problem of spectrum sharing. The main work includes the following three aspects:

(1) In the first stage, designing a secondary user utility function reflecting the needs facing the secondary network spectrum.
(2) A spectrum rental model facing these needs was designed based on Bertrand game theory, and a spectral demand adjustment algorithm of secondary base stations was also designed. This made the optimal spectrum rental quantities of secondary base stations just equal to the secondary network needs when the primary base station used Nash equilibrium pricing.
(3) In the second stage, for the communication needs of secondary users, fairly and efficiently allotting secondary base station spectrum resources based on a Nash bargaining scheme considering frequency, power and time dimensions.

## 2. System Model and Assumptions

### 2.1. Primary and Secondary Network Model

**Figure 1:**
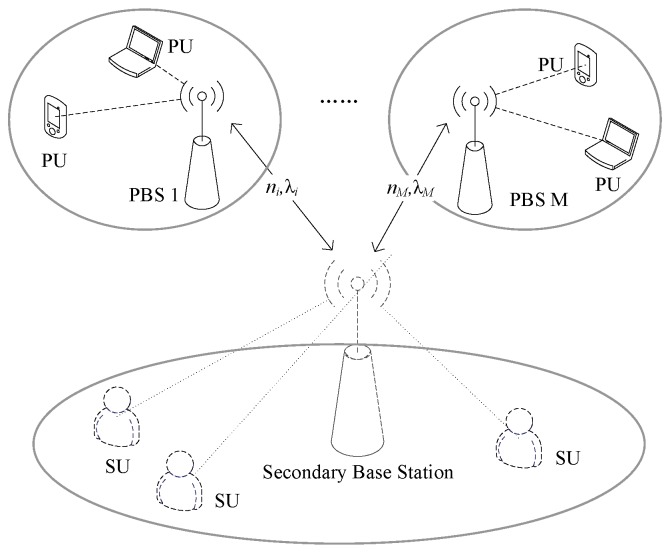
The model of multi-primary networks and a secondary network.

Suppose there is a WSN, that has *M* primary networks that can lease free spectrum to a secondary network. In order to meet the communication of secondary users, the secondary network need to lease the primary network spectrum on the basis of controlling the transmission power of secondary users. This is shown in Figure 1.

Suppose we have a secondary network comprising *N* secondary users and each secondary user i(i=1,2,…,N) can transmit channel capacity demand $R_{i}$ to the secondary station to request spectrum resources for communication services. The secondary base station collects user requirements $\overset{N}{\underset{i}{\sum }}R_{i}$ of all secondary users and decides the spectrum prices $\left(\lambda_{1},\;\lambda_{2},\ldots ,\lambda_{M}\right)$ of different primary base stations, leasing spectrum $\left(q_{1},\;q_{2},\ldots ,q_{M}\right)$ from different primary base stations, wherein $q_{i}$ refers to the number of subcarriers, assuming that all subcarriers have the same bandwidth *w*. In primary networks, each primary base station adjusts its pricing $\lambda_{k}$ continuously as seller, until they can not further increase their own income, then the spectrum market is in an equilibrium state between the spectrum price for primary base stations and the spectrum requirements for secondary base stations is $\left(\lambda_{1}^{*},\;\lambda_{2}^{*},\ldots ,\;\lambda_{M}^{*}\right)$ and $\left(q_{1}^{*},\;q_{2}^{*},\ldots ,\;q_{M}^{*}\right)$. After the secondary station pays for the spectrum resources, it would assign the corresponding spectrum resources and transmission power to each secondary user according to the needs of secondary users $\left(R_{1},R_{2},\ldots ,R_{N}\right)$ and the transmission power threshold $p^{TOTAL}$.

### 2.2. Oligopoly Market and Bertrand Game

The network model that contains many primary base stations and a single secondary base station can use oligopolistic market theory to understand the interactions of spectrum leasing process for buyers and sellers. The oligopolistic market is between the monopolistic market and the perfectly competitive market, which refers to all the commodities in the market that are controlled by a small number of sellers, each seller is able to influence the spectrum markets that contains other sellers by changing strategies such as adjusting the price or volume of goods, and sellers’ decisions are visible to each other. The Bertrand game is a kind of game in the oligopoly market, which is used to model the price competition behavior in the market, so as to analyze and get the equilibrium pricing strategy of each seller. A Bertrand game basically includes the following three elements:

(1) The player set N={1, 2, 3, …, n}, which consists of the secondary users or the primary users participating in spectrum sharing;
(2) Strategy vector a, player *i* select a strategy $a_{i}$ from its own strategy set $S_{i}$ in each game, the strategy can be the number of purchased spectrum, or transmission power, strategy vector of all players $a=(a_{1},\;a_{2},\ldots ,a_{n}$);
(3) Utility function $u_{i}\left(a_{i},\;a_{-i}\right):S\to R$, which represents the degree of benefit or satisfaction that player *i* can obtain in strategy vector $a=\left(a_{i},\;a_{-i}\right)$. Usually, $a_{-i}=\left\{a_{1},\;a_{2},\;\ldots ,\;a_{i-1},\;a_{i+1},\;\ldots ,\;a_{n}\right\}$ denotes the strategy vector of other players except *i*, and $u=\left\{u_{1},\;u_{2},\;\ldots ,\;u_{n}\right\}$ is called the utility vector.

In a Bertrand game based on an oligopoly market, the primary base stations of the primary network act as oligarchs, making decisions independently, and content of the decision is to change the price of goods. It is usually assumed that the goods of different sellers have their own characteristics, and the goods can’t be completely replaced. When the demand function of seller *i* is $q_{k}=D_{k}(\lambda_{k},\lambda_{-k})$, so *i*’s optimal pricing can be obtained by seeking extreme value income function formula (1) in the corresponding cost $C_{k}(D_{k}(\lambda_{k},\lambda_{-k}))$:

(1) $$u_{k}(\lambda_{k},\lambda_{-k})=\lambda_{k}D_{k}(\lambda_{k},\lambda_{-k})-C_{k}(D_{k}(\lambda_{k},\lambda_{-k}))$$

The primary and secondary user networks shown in Figure 1 are modeled based on the Bertrand game model. All of the primary base stations as oligarchs control the market spectrum, and each primary base station rationally adjusts the spectrum pricing in the pursuit of maximizing its own income; secondary base stations acts as buyers according to their own needs and the spectrum pricing of each primary base station to determine the amount of spectrum that they should lease from different sellers. In spectrum markets, primary and secondary base stations are competitive in the market by continuously adjusting pricing and spectrum requirements, ultimately stabilizing to a market equilibrium. The primary base stations also stabilize to a Nash equilibrium in the price competition of constantly adjusting prices.

Since the primary base stations holds licensed spectrum, they have higher spectrum access priority, so primary base stations as “leaders” determine the spectrum price λ, while the secondary base station as “followers” determine the rent number q by the spectrum prices and their demand functions. Leaders know followers will react to their policy, thus they can take the followers’ reactions into account when determining the price. That is, before the primary base station rents spectrum to a secondary base station, it will forecast the rent amount $q_{i}$ of *i* when the price is $\lambda_{i}$, and then determining the price $\lambda_{i}^{*}$ that can make their own maximum income, under this pricing, the rental amount of *i* is $q_{i}^{*}$.

**Figure 2:**
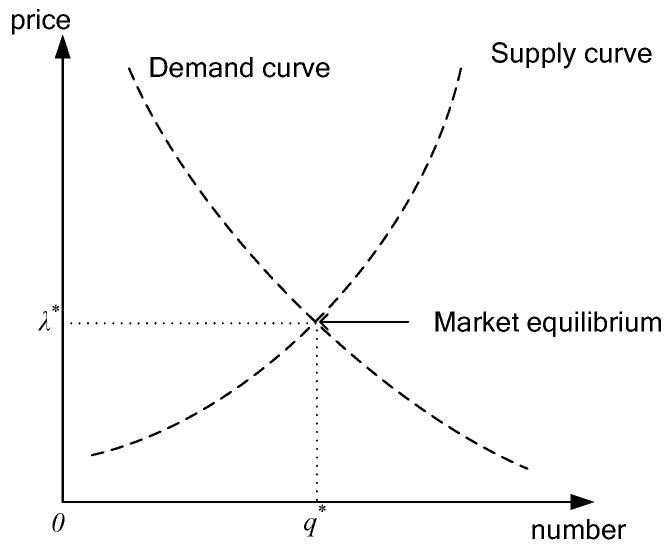
The supply and demand curves.

For this transaction process where the primary base station determines the spectrum price and the secondary user decides the number of leases, we can use a reverse induction method to solve the spectrum pricing and spectrum requirements for the equilibrium point where assuming that known the spectrum price *λ*, the supply of the primary base station is q=S(λ), and the spectrum needs of the secondary base station is q=D(λ). Usually, the supply function is a non-decreasing function with respect to the price, and the demand function is a non-increasing function with respect to the price. As shown in Figure 2, the market equilibrium $\left(\lambda^{*},q^{*}\right)$ in the supply and demand curves junction, Equation (2) can be obtained. From Equation (2), we can see that the supply of the primary base station equals the demand of all secondary base stations and $D(\lambda^{*})=S(\lambda^{*})$, that is, $\lambda^{*}=S^{-1}(q^{*})=S^{-1}(D(\lambda^{*}))$, so market equilibrium pricing and spectrum requirements of secondary users are available:

(2) $$\left\{ \begin{array}{l} q^{*}=D\left(\lambda^{*}\right)\\ q^{*}=S\left(\lambda^{*}\right) \end{array}\right.$$

## 3. The First Stage: Pricing Model Based on a Bertrand Game

Firstly, in order to quantify whether the leased spectrum satisfies the communication needs of secondary users, we need to design the utility function of the secondary base station. Secondary base stations can determine the demand function D(R, λ) of subcarriers in specific communication needs and spectrum prices by this utility function. Subsequently, modeling the pricing game of primary base stations based on the Bertrand game, it is proved that the non-cooperative game can converge to a Nash equilibrium, and the pricing method of the Nash equilibrium is used for the primary base stations.

### 3.1. The Utility Function and Demand Function of Secondary Base Stations

In order to meet the channel capacity requirements $\left(R_{1},R_{2},\ldots ,R_{N}\right)$ of all secondary users, the number of subcarriers that a secondary base station needs to hire from a primary base station is C when the primary network interference power threshold is $p^{TOTAL}$ and the average subcarrier channel gain is *h*, assuming that all secondary users use equal power $p^{TOTAL}/C$ in different subcarriers. According to Shannon’s theorem, C satisfies Equation (3), because the secondary users use M-quadrature amplitude modulation [24], so, *η* = −1.5/(*δ_z_*^2^·ln(5*BER_ij_*)). By solving Equation (3), we can obtain the total available subcarriers that a secondary base station needs, indicated by Equation (4), where *δ* = *p^TOTAL^*·|*h*|^2^·*η*, W(·) is the Lambert-W function.

(3) $$R=\sum_{i=1}^{N}R_{i}=C\cdot w\cdot \log_{2}(1+\frac{p^{TOTAL}}{C}|h{|}^{2}\eta )(bit/s)$$

(4) $$C=-\frac{\delta }{w\cdot \delta \cdot W[-2\exp(-R/(\delta \cdot w))\cdot R/(\delta \cdot w)]/R+1}$$

So, the utility function of secondary base station is designed as follows:

(5) $$u_{SBS}(q)=u_{1}+u_{2}+u_{3}$$

$u_{1}=\mu -\alpha {\left(C-\sum_{k=1}^{M}q_{k}\right)}^{2}$, $u_{2}=-\frac{1}{2}(\sum_{k=1}^{M}q_{k}^{2}+2v\sum_{j\neq k}^{M}q_{k}q_{j})$, $u_{3}=-2v\sum_{k=1}^{M}q_{k}\lambda_{k}$ $q=\left\{q_{1},q_{2},\ldots ,q_{M}\right\}$ represents the number of subcarriers that rent from different primary base stations. The utility function consists of three parts:

(1) $u_{1}$ represents the utility that leased spectrum can provide. When the rented spectrum is just equal to the spectrum requirements, $u_{1}$ can obtain the maximum benefit. If the leased spectrum is less than the spectrum requirements, the quality that the communication of secondary networks needs can’t be guaranteed. If the leased spectrum is more than the spectrum requirements, this would result in excess spectrum and reduced resource utilization. The preference coefficient α reflects the desirability that a secondary base station meets its demand. In addition, to ensure that the utility value of this part is positive, setting μ(μ>0) is the upper limit of its utility value.
(2) $u_{2}$ describes the mutual substitutability of the spectrum sold by different primary base stations. The Bertrand game assumes the rental spectrum of different primary base stations can’t be completely replaced, therefore, a spectrum replacement rate *v* is introduced to consider the spectrum substitutability of different primary base stations. The meaning of replacement rate v∈[−1.0,1.0], that is, when v=0.0, the secondary user can’t switch between different primary base station spectra; when v=1.0, the secondary user can switch between different spectra; when v<0, the user spectrum sharing needa to use complementary spectrum, that is, when a secondary user uses a spectrum of the primary base station, it needs to lease one or more other spectra of primary base stations together.
(3) $u_{3}$ represents the cost that must be paid for the subcarriers $\left\{q_{1},q_{2},\ldots ,q_{M}\right\}$ of the primary base station.

When given the price of spectrum $\lambda =(\lambda_{1},\lambda_{2},\ldots ,\lambda_{M})$, the following conclusions are established:

**Theorem** **1.** *When the utility function of the secondary base station is given by Equation (5), the corresponding demand function that the subcarriers share is:*

(6) $$D_{k}(\lambda )=D_{1}\cdot \lambda_{k}+D_{2}(\lambda_{-k}),k=1,2,...,M$$

$$D_{1}=\frac{1}{1-v}[\frac{2\alpha +v}{2\alpha \cdot M+1+v\cdot (M-1)}-1],\;D_{2}(\lambda_{-k})=\frac{1}{1-v}\cdot [2\alpha \cdot C-(2\alpha +v)\frac{2\alpha \cdot M\cdot C-\sum_{j\neq k}\lambda_{j}}{2\alpha \cdot M+1+v(M-1)}]$$

**Proof.**  Since the utility function (5) is a concave function, namely, $\frac{\partial^{2}u_{SBS}(q)}{\partial q_{i}{}^{2}}<0$, we just need to get the maximum value of the function to get $q_{k}=D_{1}\lambda_{k}+D_{2}(\lambda_{-k}),(k=1,2,...,k)$, which proves the theorem. ☐

### 3.2. The Utility of the Primary Base Station and the Bertrand Game

For the network model which has several or more primary networks and only one secondary network, we know that the supply of the primary base station *k* is equal to the market demand which we called $D_{k}(\lambda )$ when the market is proportionate, so the corresponding utility is:

(7) $$u_{k}^{PBS}(\lambda_{k},\lambda_{-k})=\lambda_{k}\cdot D_{k}(\lambda_{k},\lambda_{-k})-\beta_{k}\cdot D_{k}(\lambda_{k},\lambda_{-k})$$

The loss coefficient of $\beta_{k}$ stands for the loss caused by the primary base station *k* in the process of renting. In the market of this spectrum, spectrum pricing of the primary base station *k* is called $\lambda_{k}$; it will not only be influenced by other primary base stations’ pricing, but it also affects the pricing of other primary base stations. To deal with this problem, we can build a Bertrand competition to study the price competition among different primary base stations. For the corresponding Bertrand competition Γ=〈M,S,u〉, let:

(1) M represents the set of game players. This set is composed of primary base stations, such as {1, 2, …, *M*}.
(2) $\lambda_{k}\in S_{k}$ represents a player’s strategy. The primary base station sets the renting price $S_{k}=\left\{\lambda_{k}|\lambda_{k}>\beta_{k},\lambda_{k}\in R^{+}\right\}$, *k* ∈ M of subcarriers of per unit.
(3) $\mu_{k}\left(\lambda_{k},\lambda_{-k}\right)$ represents a payoff function. The primary base station *k* determines the available utility that spectrum pricing $\lambda_{k}$ could acquire, calculated by the formula $\mu_{k}^{PBS}\left(\lambda_{k},\lambda_{-k}\right)$.

The primary base station *k*, as a player in the Bertrand games constantly adjusts its pricing, in satisfying certain conditions, it can eventually be stable in a state of equilibrium, which is called a Nash equilibrium (NE).

**Definition** **1** (Nash Equilibrium). The Nash equilibrium of the strategic form game is a strategy vector $\lambda^{*}=\left(\lambda_{k}^{*},\;\lambda_{-k}^{*}\right)$, $\lambda^{*}$ satisfies:

(8) $$u_{i}\left(\lambda_{k}^{*},\lambda_{-k}^{*}\right)\geq u_{k}\left(\lambda_{k},\lambda_{-k}^{*}\right),\;\forall k\in M,\;\forall \lambda_{k}\in S_{k},\;\lambda_{-k}^{*}\in S_{-k}$$

This definition shows that no player can unilaterally change its own strategy to obtain a greater incentive in a Nash equilibrium, so, when other players choose a Nash equilibrium strategy, the best strategy of the player can choose is the Nash equilibrium strategy, that is:

(9) $$\lambda_{k}^{*}\in B_{k}(\lambda_{-k}^{*})=\arg\max_{a_{k}\in S_{k}}\;u_{k}(\lambda_{k},\lambda_{-k}^{*}),\;\forall k\in M$$

Herein the function $B_{k}\left(\cdot \right)$ is called the optimal response function. However, a Nash equilibrium doesn’t always exist in any form of strategy. The existence condition of a Nash equilibrium is given in [25].

**Lemma** **1.** *For any player* k∈M *in the strategic form of game* Γ=〈M,S,u〉, *if the policy set* $S_{k}$ *is a convex compact subset of the Euclidean space, the utility function* $u_{k}$ *is to be concave and continuous on the policy set* $S_{k}$ *then the Nash equilibrium of this game exists*.

**Theorem** **2.** *For Bertrand game* Γ=〈M,S,u〉 *there exists a Nash equilibrium*.

**Proof.**  A Bertrand competition in normal form is a structure Γ=〈M,S,u〉, where a player’s strategy is a nonempty convex subset $\left\{\lambda_{k}|\lambda_{k}\rangle \beta_{k},\lambda_{k}\in R^{+}\right\}$. We can prove that $\mu_{k}^{PBS}$ is a concave function by Equations (6) and (7). By Theorem 2 and Equation (9), we can find that there is a Nash equilibrium $\left(\lambda_{1}^{*},\lambda_{2}^{*},\ldots ,\lambda_{M}^{*}\right)$ in this game, and $\lambda_{k}^{*}=\arg\max_{\lambda_{k}\in S_{k}}\;u_{k}^{PBS}(\lambda_{k},\lambda_{-k}^{*}),\forall k\in M$, which proves the theorem. ☐

This means that when all parameters are known in the network, we can get a Nash equilibrium pricing strategy (Equation (11)) of a primary base station by Equation (6) and linear Equation (10) and the amount of subcarrier that each secondary base station rents from the primary base station could be solved by its demand function (Equation (6)):

(10) $$\frac{\partial u_{k}^{PBS}(\lambda )}{\partial \lambda_{k}}=0,k=1,2,...,M$$

(11) $$\lambda_{k}^{*}=\underset{\lambda_{k}\in S_{k}}{\arg\max}\;u_{k}^{PBS}(\lambda_{k},\lambda_{-k}^{*})=\beta_{k}/2-D_{2}(\lambda_{-k}^{*})/2D_{1},\forall k=1,2,...,M$$

For example, in the situation of two primary base stations, the corresponding linear equation is:

(12) $$\left\{ \begin{array}{l} \frac{1}{1-v}[2\alpha \cdot C-(2\alpha +v)\frac{2\alpha \cdot M\cdot C-\lambda_{2}}{2\alpha \cdot M+1+v(M-1)}]+\frac{2\lambda_{1}-\beta_{1}}{1-v}[\frac{2\alpha +v}{2\alpha \cdot M+1+v\cdot (M-1)}-1]=0\\ \frac{1}{1-v}[2\alpha \cdot C-(2\alpha +v)\frac{2\alpha \cdot M\cdot C-\lambda_{1}}{2\alpha \cdot M+1+v(M-1)}]+\frac{2\lambda_{2}-\beta_{2}}{1-v}[\frac{2\alpha +v}{2\alpha \cdot M+1+v\cdot (M-1)}-1]=0 \end{array}\right.$$

### 3.3. Dynamic Bertrand Game

Different primary base stations in different primary networks can’t monitor the earnings that each other has acquired and also know nothing about the current strategies of others, so they need to obtain optimal pricing by designing a corresponding distributed program. We provide a distributed program based on dynamic Bertrand competition, which iterates the Bertrand competition process repeatedly to reach optimal pricing. For every iteration named *t* and each primary base station named *i*, when the spectrum pricing of *i* is $\lambda_{k}\left[t\right]$, other primary base stations’ pricing will be $\lambda_{k}\left[t\right]$. When we enter the next t+1 iteration, the primary base station *k* adjusts the spectrum pricing of this iteration with a different update strategy to approach Nash equilibrium closer and closer using the different information acquired, where:

(1) The update strategy of pricing can use the following formula if the primary base station can obtain other players’ historical strategy during the last iteration:
  (13) $$\lambda_{k}[t+1]=B_{k}(\lambda_{-k}[t])=\underset{\lambda_{k}\in S_{k}}{\arg\max}\;u_{k}^{PBS}(\lambda_{k},\lambda_{-k}[t]),\forall k\in M$$
(2) The following learning strategy will be used when the primary base station can only study local information, where $\sigma_{k}$ means the step size of each update:
  (14) $$\lambda_{k}[t+1]=\lambda_{k}[t]+\sigma_{k}\cdot (\frac{\partial u_{k}^{PBS}}{\partial \lambda_{k}})$$

The marginal revenue $\frac{\partial u_{PBS}}{\partial \lambda }$ of Equation (14) can be calculated by Equation (15). When setting a suitable threshold *τ* and Equation (16) is established, with the increase of the number of iterations and when the utility value of the primary user is close to stable, we can argue that the dynamic Bertrand game can reach the equilibrium state. Moreover, it also can use Equation (17) to calculate the marginal revenue by observing the margin of their utility’s change when a minute pricing adjustment occurs. Let ε represent the margin of each minute adjustment, it will stop updating when the conditions of Equation (18) are satisfied:

(15) $$\frac{\partial u_{k}^{PBS}(\lambda )}{\partial \lambda_{k}}=D_{k}(\lambda )+(\lambda_{k}-\beta_{k})\cdot \frac{\partial D_{k}(\lambda )}{\partial \lambda_{k}}=D_{k}(\lambda )+(\lambda_{k}-\beta_{k})\cdot D_{1}$$

(16) $$D_{k}(\lambda )+(\lambda_{k}-\beta_{k})\cdot D_{1}<\tau$$

(17) $$\frac{\partial u_{PBS}}{\partial \lambda }\approx \frac{u_{PBS}(\lambda [t]+\varepsilon )-u_{PBS}(\lambda [t]-\varepsilon )}{2\varepsilon }$$

(18) $$u_{PBS}(\lambda [t]+\varepsilon )-u_{PBS}(\lambda [t]-\varepsilon )<\tau$$

### 3.4. Stability and Complexity Analysis of Dynamic Game

By analysis of the stability and complexity of a dynamic game, we can definite whether this dynamic game could reach a Nash equilibrium when it converges to a stable state gradually and how to converge to stable state quickly. We can see the two update strategies in Equations (13) and (14) as two different self-mapping functions, so we can learn from the Routh-Hurwitz criterion that this self-mapping function is stable if and only if all its eigenvalues *ξ_i_* are located in the unit circle of a complex plane, that means |*ξ_i_*|<1. We define a Jacobian matrix shown in (19) in order to obtain the solution set of eigenvalues of the self-mapping function:

(19) $$\left[\begin{array}{cccc} \frac{\partial \lambda_{1}[t+1]}{\partial \lambda_{1}[t]} & \frac{\partial \lambda_{1}[t+1]}{\partial \lambda_{2}[t]} & \cdots & \frac{\partial \lambda_{1}[t+1]}{\partial \lambda_{M}[t]}\\ \frac{\partial \lambda_{2}[t+1]}{\partial \lambda_{1}[t]} & \frac{\partial \lambda_{2}[t+1]}{\partial \lambda_{2}[t]} & \cdots & \frac{\partial \lambda_{2}[t+1]}{\partial \lambda_{M}[t]}\\ \vdots & \vdots & \ddots & \vdots \\ \frac{\partial \lambda_{N}[t+1]}{\partial \lambda_{1}[t]} & \frac{\partial \lambda_{N}[t+1]}{\partial \lambda_{2}[t]} & \cdots & \frac{\partial \lambda_{M}[t+1]}{\partial \lambda_{M}[t]} \end{array}\right]$$

(20) $$\left[\begin{array}{cccc} 0 & \chi_{1} & \cdots & \chi_{1}\\ \chi_{1} & 0 & \cdots & \chi_{1}\\ \vdots & \vdots & \ddots & \vdots \\ \chi_{1} & \chi_{1} & \cdots & 0 \end{array}\right],\chi_{1}=\frac{2\alpha +v}{2[2\alpha \cdot (M-1)+v\cdot (M-1)+1-v]}$$

(21) $$\left[\begin{array}{cccc} 1+\frac{2\sigma_{1}(\chi_{2}-1)}{1-v} & \frac{\sigma_{1}\chi_{2}}{1-v} & \cdots & \frac{\sigma_{1}\chi_{2}}{1-v}\\ \frac{\sigma_{2}\chi_{2}}{1-v} & 1+\frac{2\sigma_{2}(\chi_{2}-1)}{1-v} & \cdots & \frac{\sigma_{2}\chi_{2}}{1-v}\\ \vdots & \vdots & \ddots & \vdots \\ \frac{\sigma_{M}\chi_{2}}{1-v} & \frac{\sigma_{M}\chi_{2}}{1-v} & \cdots & 1+\frac{2\sigma_{M}(\chi_{2}-1)}{1-v} \end{array}\right],\chi_{2}=\frac{2\alpha +v}{2\alpha \cdot M+1+v(M-1)}$$

At first, the dynamic game is based on Equation (13) and its Jacobian matrix is Equation (20). By calculating the steps described above, we can get its eigenvalues: $\xi_{1}=\ldots =\xi_{M-1}=-\chi_{1}$, $\xi_{M}=\left(M+1\right)\cdot \chi_{1}$. Since the replacement rate ν∈[−1.0,1.0], we can know that the molecule of all eigenvalues is less than the denominator, that is $\left|\xi_{k}\right|<1,\left(k=1,\;2,\ldots ,\;M\right)$, so the dynamic game of the self-mapping function (13) is stable.

The matrix shown in (21) is the Jacobian matrix of the dynamic game presented in (14). Let $\sigma_{1}=\sigma_{2}=\sigma_{M}=\sigma$, we can obtain its relevant eigenvalues: $\xi_{1}=\ldots =\xi_{M-1}=1+\left[\sigma \left(\chi_{2}-2\right)\right]/\left(1-v\right)$, $\xi_{M}=1+\left\{\sigma \left[\left(M+1\right)\chi_{2}-2\right]\right\}/\left(1-v\right)$. Because $-\left(2M-1\right)/\left(M-1\right)<\;\chi_{2}-2<0$ and $-2<\left(M+1\right)\chi_{2}-2<0$, so by finding the solution of inequality $\left|\xi_{k}\right|<1,\left(k=1,\;2,\ldots ,\;M\right)$, we can come to a conclusion that the sufficient condition for a dynamic game to be stable is obtained by updating the step size σ of dynamic game that must satisfy constraint: σ∈(0,(1−v)· (2M−2)/(2M−1)),(M>1).

It can be seen from the above stability analysis that the dynamic game update strategy of (13) can converge to the Nash equilibrium according to the optimal response value. The convergence rate of the dynamic game update strategy in (14) is highly dependent on the update step, and the complexity of the model depends on the convergence rate and number of iterations of the dynamic game, so the update step is the key to the complexity of the model. In the later simulation experiments, we will further discuss the effect of the update step on the convergence speed of the dynamic game to ensure the lowest model complexity.

### 3.5. Spectrum Requirement Adjustment and System Scheduling

In the model of spectrum pricing and spectrum allocation which are both based on Bertrand competition, when the subcarrier rent in the state of market equilibrium is lower than the demand of subcarriers in secondary networks, rental subcarriers will not satisfy the spectrum requirements of the secondary network.

**Figure 3:**
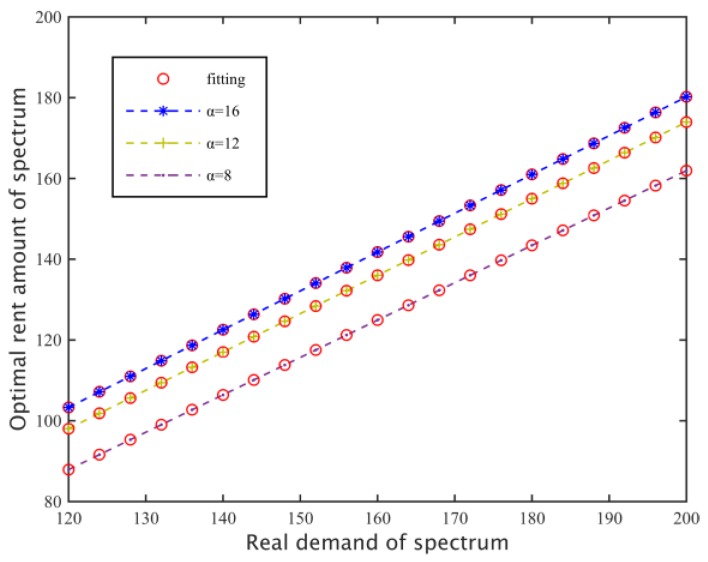
The relationship between spectrum demand and optimal rent number.

From Figure 3, we can learn that when we have set preference factor, replacement rate and loss coefficient, then both the real spectrum requirements and the optimal rent total amount $q^{*}=\sum_{k=1}^{M}D_{k}(\lambda^{*})$ will linearly increase, so we can do the next two steps. Firstly, secondary base stations could present their actual spectrum requirements to the primary base station in advance and then by the optimal pricing that the primary network has formulated, we could define the relationship between the actual spectrum requirements and the amount of rental subcarriers. Secondly, in order to acquire spectrum resources that could fulfill the demands of the secondary networks, we can adjust the spectrum requirements again based on the first step. That means, the secondary base stations send actual requirements $C+\varepsilon^{\prime }$ and $C-\varepsilon^{\prime }$, which have undergone a minute adjustment to each primary base station, and then calculate the corresponding total amount of rental subcarriers $q_{total}^{*}\left(C+\varepsilon^{\prime }\right)$ and $q_{total}^{*}\left(C-\varepsilon^{\prime }\right)$ by the requirement function (6) after each base station has returned the optimal pricing $\lambda^{*}\left(C+\varepsilon^{\prime }\right)$ and $\lambda^{*}\left(C-\varepsilon^{\prime }\right)$ under Nash Equilibrium conditions. Secondary base stations find the solution set of slopes which indicate the linear relationship between their requirements and the amount of rent subcarriers by Equation (22), and then determine which virtual spectrum requirement C′ to send to the primary base station using Equation (23). Through lots of experiments, we can know that the amount of spectrum rental subcarriers based on the subcarrier requirements could satisfy the spectrum requirements of the secondary networks:

(22) $$slope\approx \frac{q_{sum}^{*}(C+\varepsilon^{\prime })-q_{sum}^{*}(C-\varepsilon^{\prime })}{2\varepsilon^{\prime }}$$

(23) $$C^{\prime }=(C-q_{sum}^{*}(C-\varepsilon^{\prime }))/slope+C-\varepsilon^{\prime }$$

**Figure 4:**
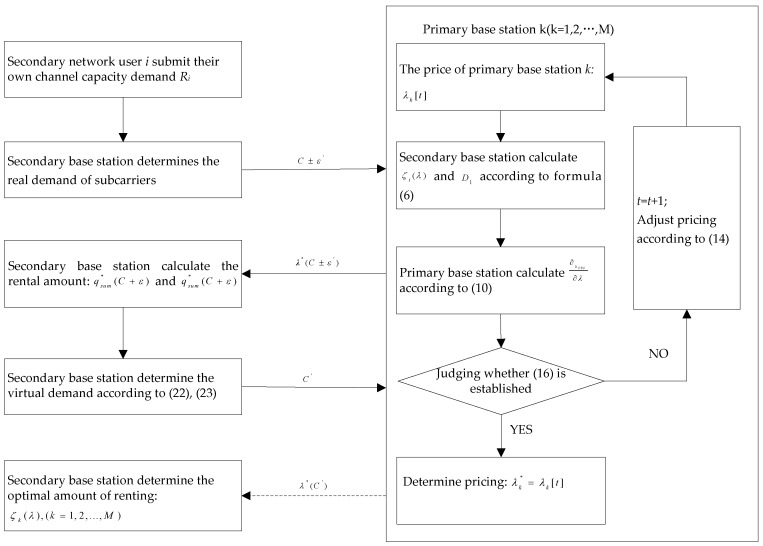
Spectrum pricing and allocation scheduling.

In summary, when the primary base station is based on the local information of dynamic spectrum pricing, the scheduling process of first stage is as shown in Figure 4.

## 4. The Second Stage: Cooperation Spectrum Sharing

Secondary base station can obtain the set SC of subcarriers in a total number $\sum_{k=1}^{M}D_{k}(\lambda )$ by paying for a number of primary base stations. This is used in the communication services of secondary users in the secondary network, so secondary base stations need to solve how to assign a set of subcarriers for each secondary user based on the channel capacity requirements $\left(R_{1},R_{2},\ldots ,R_{N}\right)$ and the transmission power $p_{ij}$ of each subcarrier *j*, and ensure not causing interference for the primary network communication. According to the characteristics of the problem, we can model and solve the problem by using the bargaining game model of cooperative game theory.

### 4.1. Spectrum Sharing Based on a Bargaining Game

Secondary users in a secondary network can fairly and efficiently share available spectrum resources through cooperative games. Bargaining games, as a kind of a cooperative game, can offer game players a win-win and fair solution:

**Definition** **2** (Bargaining game and bargaining solution)**.** *The game $\Gamma =\left(N,S,U,u^{0}\right)$ is called the bargaining game, if **U** is a closed convex subset of **R**^N^, and there is at least one feasible payoff vector, for any I, $u_{i}\geq u_{i}^{0}$ that exists. A bargaining scheme is a function that maps the bargaining problem to the only feasible payoff vector:* $f\left(U,u^{0}\right):\;U\to u^{*}$.

According to the bargaining game definition, we can regard secondary users in secondary networks as game players that reach a cooperative agreement, and we can use a bargaining game to set up a module for the secondary base station spectrum resource allocation problem. The corresponding bargaining game formula is $\Gamma =\left(N,S,U,u^{0}\right)$:

(1) The set of game players: secondary user set {1, 2,…, i,…, N};
(2) Player strategy $a_{i}=\left(SC_{i},p_{i}\right)\in S_{i}$: the secondary user *i* asks the secondary base station for communication subcarrier set $SC_{i}$. The power transmission of every subcarrier is $p_{i}=\left\{p_{ij}\right\},\;(j\in SC_{i}$);
(3) Payoff function $u_{k}\left(a_{k},a_{-k}\right)$: The user *i* uses power $p_{ij}$ to transport information in the corresponding subcarrier of the subcarrier set $SC_{i}$. It can obtain channel capacity $\underset{j\in SC_{i}}{\sum }w\cdot log_{2}\left(1+p_{ij}{\left|h_{ij}\right|}^{2}\cdot \eta \right)$.
(4) Payoff vector is $u^{0}$ when it doesn’t reach a cooperation agreement: The demand of channel capacity of the secondary user is (R_1_, R_2_, …, R*_N_*);

Thus, the problem of subcarriers and power allocation of secondary base station can be transformed into a feasible payoff vector problem in cooperative spectrum communion based on a bargaining game. The bargaining solution is considered a kind of selection criteria of the optimal feasible payoff vector. The Nash bargaining solution (NBS) is a kind of general resource sharing bargaining solution.

Not only can it satisfy the secondary user’s needs, but also it can ensure the fairness of resource allocation to the greatest extent. Therefore, according to the Nash bargaining solution, we can define the allocation problem of subcarrier set $SC_{i}$ and power transmission $p_{ij}$ as the nonlinear programming problem shown in Equation (24) in order to fairly and efficiently assign spectrum resources in the secondary network. In the equation, $r_{ij}=w\cdot log_{2}\left(1+p_{ij}{\left|h_{ij}\right|}^{2}\cdot \eta \right)$ represents the fact that secondary user *i* can obtain channel capacity by using subcarrier *j* to transfer. The constraint (1) makes it possible for every the secondary user to get higher channel capacity than demanded. In order to avoid affecting the primary network communication, the condition (2) controls that the power transmission of the secondary user network is less than the interference power threshold $p^{TOTAL}$. The condition (3) ensures that subcarriers assigned for all the secondary users will be in the subcarrier set rented by the secondary base station:

(24) $$\begin{array}{l} \max\prod_{i=1}^{N}\left(\sum_{j\in SC_{i}}w\cdot \log_{2}(1+p_{ij}|h_{ij}{|}^{2}\cdot \eta )-R_{i}\right)\\ s.t.(1)\sum_{j\in SC_{i}}w\cdot \log_{2}(1+p_{ij}|h_{ij}{|}^{2}\cdot \eta )\geq R_{i}\\ (2)\sum_{i=1}^{N}\sum_{j\in SC_{i}}p_{ij}\leq p^{TOTAL}\\ (3)\sum_{i=1}^{N}SC_{i}\leq SC\\ (4)SC_{i},p_{ij}\in R^{+},\forall i=1,2,...,N,j\in SC_{i} \end{array}$$

### 4.2. The Optimal Allocation of Subcarriers, Power and Time

For nonlinear programming Equation (24), the general solution is to list all subcarrier sets and transmission power levels. However, for communication systems that allow a time sharing strategy, the exhaustive method will lead to inestimable computing resource consumption because the full range extends from the finite set to the infinite set. We can reduce the complexity of solving the problem through introducing time allocation scheduling. Namely, the secondary base station used all subcarriers for a communication service for a certain user. Therefore, the allocation problem of subcarrier set and transmission power (24) can be translated into the allocation problem of transmission time and transmission power (25). In the equation, $t_{i}$ represents that the time which all subcarriers are assigned by the secondary base station is used for the secondary user *i* is the proportion accounting for the entire scheduling cycle. Consequently, the actual transmission time of the secondary user *i* is $t_{i}\cdot T$, and T is the total time of scheduling period:

(25) $$\begin{array}{l} \max\prod_{i=1}^{N}\left(t_{i}\cdot \sum_{j\in SC}w\cdot \log_{2}(1+p_{ij}\cdot |h_{ij}{|}^{2}\cdot \eta )-R_{i}\right)\\ s.t.(1)t_{i}\cdot \sum_{j\in SC}w\cdot \log_{2}(1+p_{ij}\cdot |h_{ij}{|}^{2}\cdot \eta )\geq R_{i}\\ (2)\sum_{j\in SC}p_{ij}\leq p^{TOTAL},\forall i=1,2,...,N\\ (3)\sum_{i=1}^{N}t_{i}\leq 1\\ (4)p_{ij}\in R^{+},\forall i=1,2,...,N,j\in SC \end{array}$$

**Theorem** **3.** A feasible solution for the nonlinear programming problem (25) exists only when $\sum_{i=1}^{N}R_{i}/r_{i}^{*}\leq 1$ In the formula, the optimal power allocation strategy and time allocation strategy are:

(26) $$p_{ij}^{*}={\left[\frac{1}{\left|SC\right|}\cdot (p^{TOTAL}+\sum_{l\in SC}\frac{1}{|h_{il}{|}^{2}\cdot \eta })-\frac{1}{|h_{ij}{|}^{2}\cdot \eta }\right]}^{+},i=1,2,...,N,j\in SC$$

(27) $$t_{i}^{*}=\frac{1}{N}\cdot (1-\sum_{l=1}^{N}\frac{R_{l}}{r_{i}^{*}})+\frac{R_{i}}{r_{i}^{*}},i=1,2,...,N$$

where [x]^+^ represents max{x,0}, and $r_{i}^{*}=\sum_{j\in \mathrm{SC}}w\cdot log_{2}\left(1+p_{ij}^{*}\cdot {\left|h_{ij}\right|}^{2}\cdot \eta \right)$.

**Proof.**  The optimal power allocation is considered firstly. For the feasible time allocation strategy $t_{i}^{*}$ given $p=\left(p_{ij}^{*}\right)\;\left(i=1,2,..,N,\;j\in SC\right)$ is the optimal power allocation strategy for (25) only when $p_{ij}^{*}$ is also the optimal solution for programming problem (28):

(28) $$\begin{array}{l} \max\sum_{j\in SC}w\cdot \log_{2}(1+p_{ij}\cdot |h_{ij}{|}^{2}\cdot \eta )\\ s.t.\sum_{j\in SC}p_{ij}\leq p^{TOTAL},\forall i=1,2,\cdots ,N \end{array}$$

So, the optimal power allocation problem for (25) can be obtained by the Lagrange Multiplier Approach solving programming problem (27). The corresponding Lagrange function is:

(29) $$L(p,\mu )=\sum_{j\in SC}w\cdot \log_{2}(1+p_{ij}\cdot |h_{ij}{|}^{2}\cdot \eta )-\sum_{i=1}^{N}\mu_{i}(\sum_{j\in SC}p_{ij}-p^{TOTAL})$$

In the equation, $\mu =\left(\mu_{1},\;\mu_{2},\;\ldots ,\;\mu_{N}\right)$ is Lagrange multiplier, so:

(30) $$\frac{\partial L(p,\lambda )}{\partial p_{ij}}=\frac{w\cdot |h_{ij}{|}^{2}\cdot \eta }{\ln2\cdot (1+p_{ij}\cdot |h_{ij}{|}^{2}\cdot \eta )}-\mu_{i}$$

(31) $$\frac{\partial L(p,\mu )}{\partial \mu_{i}}=\sum_{j\in SC}p_{ij}-p^{TOTAL}$$

☐

We can apply the KKT condition to get Equations (32) and (33). The optimal power allocation strategy (26) can be achieved by substituting Equation (33) into Equation (32):

(32) $$p_{ij}^{*}={\left(\frac{w}{\ln2\cdot \mu_{i}^{*}}-\frac{1}{|h_{ij}{|}^{2}\cdot \eta }\right)}^{+}$$

(33) $$\frac{1}{\mu_{i}^{*}}=\frac{\ln2}{w\cdot |SC|}\left\{p^{TOTAL}+\sum_{l\in SC}\frac{1}{|h_{il}{|}^{2}\cdot \eta }\right\}$$

Then, the existence of the feasible solution should be considered. The existence of the optimal power allocation strategy doesn’t mean that the feasible solution for the programming problem (25) exists. Based on the optimal allocation strategy (26), the feasible solution for (25) exists when the constraint conditions (1) and (3) of (25) are satisfied. Therefore, through putting the optimal power allocation strategy into (1) and (3), we can get the necessary and sufficient condition for the existence of feasible solution $\sum_{i=1}^{N}\frac{R_{i}}{r_{i}^{*}}\leq 1$:

(34) $$\begin{array}{l} \max\prod_{i=1}^{N}\left(t_{i}\cdot r_{i}^{*}-R_{i}\right)\\ s.t.\sum_{i=1}^{N}t_{i}\leq 1 \end{array}$$

(35) $$\begin{array}{l} \max\sum_{i=1}^{N}\ln(t_{i}\cdot r_{i}^{*}-R_{i})\\ s.t.\sum_{i=1}^{N}t_{i}\leq 1 \end{array}$$

Lastly, based on the optimal allocation, we consider the optimal time allocation. Let $r_{i}^{*}=\sum_{j\in SC}w\cdot log_{2}\left(1+p_{ij}^{*}\cdot {\left|h_{ij}\right|}^{2}\cdot \eta \right)$, and we can achieve the programming problem (34) by putting $p=\left(p_{ij}^{*}\right)$ into Equation (25). We can solve the programming problem (35) through logarithmic transformation of Equation (34) because f(x)=ln(x) is a monotonically increasing concave function in domain. Meanwhile, the Lagrange Multiplier Approach is used for solving the problem. The corresponding Lagrange function is:

(36) $$L(t,\varphi )=\sum_{i=1}^{N}\ln(t_{i}\cdot r_{i}^{*}-R_{i})-\varphi \cdot (\sum_{i=1}^{N}t_{i}-1)$$

Here φ is the Lagrange multiplier. By applying the KKT condition, we can get the equations:

(37) $$\frac{\partial L(t,\varphi )}{\partial t_{i}}=\frac{r_{i}^{*}}{t_{i}\cdot r_{i}^{*}-R_{i}}-\varphi =0$$

(38) $$\frac{\partial L(t,\varphi )}{\partial \varphi }=\sum_{i=1}^{N}t_{i}-1=0$$

**Figure 5:**
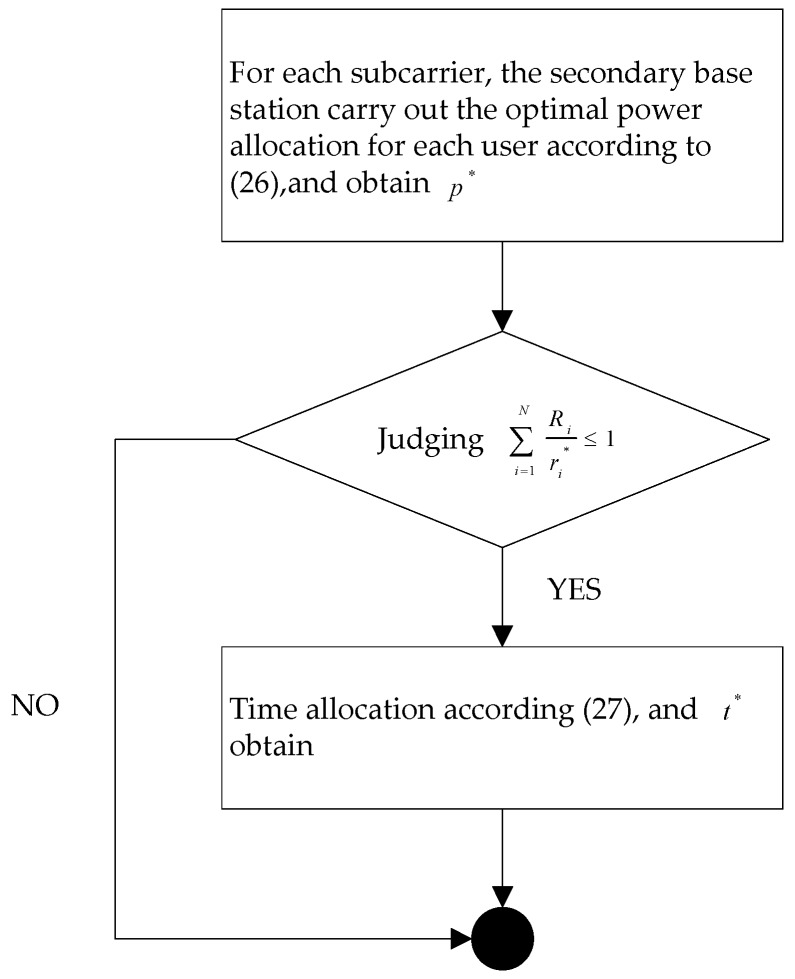
Spectrum pricing and allocation scheduling.

The optimal time allocation can be achieved through solving Equations (37) and (38). Therefore, the corresponding subcarriers and power optimal allocation process can be expressed as shown in Figure 5.

## 5. Simulation

The simulation uses two primary networks, setting the primary network interference power threshold as *p^TOTAL^* = 50 mW. The bandwidth of each subcarrier *w* = 25 kHz, thermal noise level *δ_Z_*^2^ = 10^−11^ W, desired BER = 10^−2^, and the simulation channel is modeled by Rayleigh fading and the loss factor is 3. Suppose that two secondary users are located at a distance of 200 m within the range of the base station in the secondary network, and their respective channel capacity requirements are R_1_ = 2 Mb/s, R_2_ = 3 Mb/s. Considering the path loss of the channel at the boundary (when the distance from the base station is 200 m), when the subcarrier demand is 144 by Equation (4) or enumeration, a channel capacity of 5.0 Mb/s can be provided.

### 5.1. Spectrum Pricing and Allocation 

#### 5.1.1. Preference Coefficient and Rate of Substitution 

**Figure 6:**
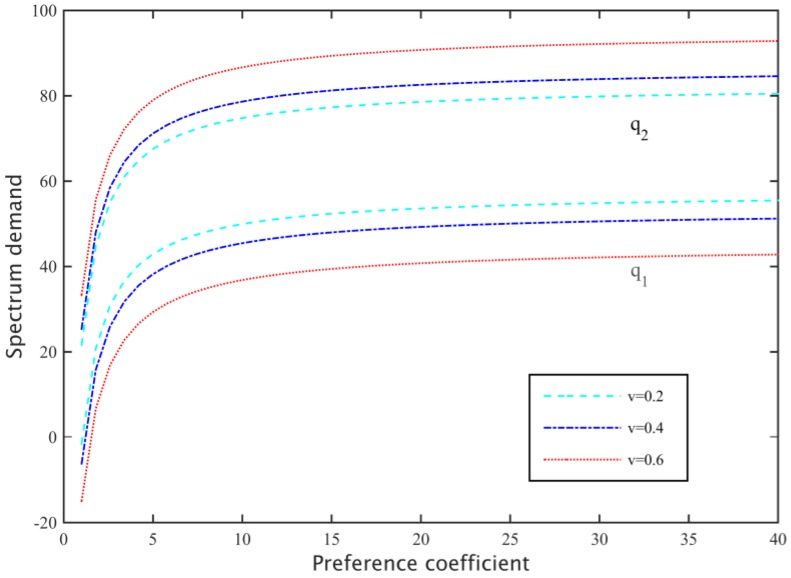
Influence of preference coefficient on spectrum demand.

After setting the spectrum pricing of two primary base station as *p*_1_ = 460, *p*_2_ = 440, the effects of different preference coefficients *α* and substitution rates *v* on the rent number of subcarriers in the secondary networks are studied. As shown in Figure 6, the secondary base station rents the number of subcarriers whose price is significantly lower than the higher price. If the degree of preference of the secondary base station to satisfy the demand is greater, it will tend to hire more subcarriers to meet the needs of secondary networks. However, when the number of subcarriers meets the demand, the preference coefficient increase that influences the number of subcarriers rented becomes gradually smaller. In addition, Figure 6 shows that substitution rate is smaller, and the switching difficulty that secondary users in the spectrum of different primary base stations experience will be bigger, thus they have to rent more expensive subcarriers; when the substitution rate is higher, secondary users are more likely to rent relatively cheap subcarriers.

#### 5.1.2. The Influence of other Primary Base Stations on Spectrum Pricing 

**Figure 7:**
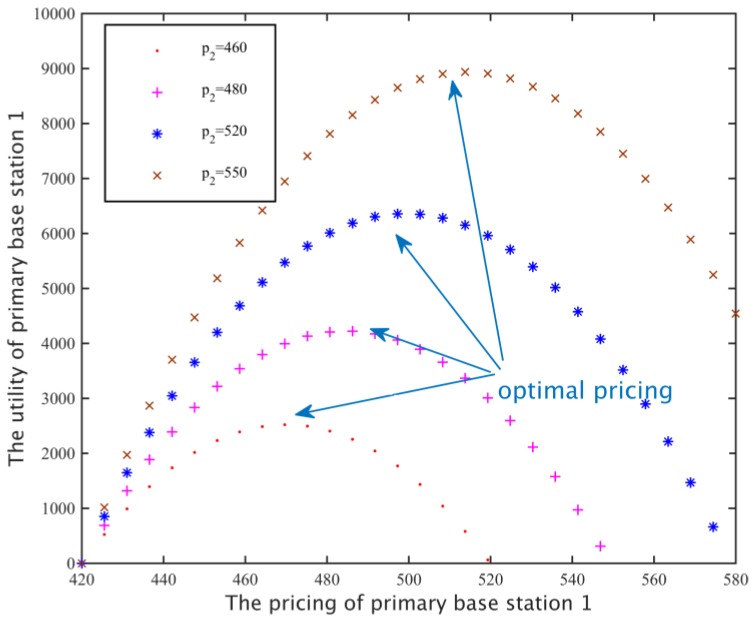
Utility function of a primary base station.

According to the results in Section 5.1.2, we set the preference coefficient *α* = 12, and the replacement rate of *v* = 0.4 and set different spectrum pricing values of the primary base station 2: *λ*_2_ = 460, 480, 520, 550, and then we examine the relationship between the spectrum pricing and revenue of the primary base station 1. As shown in Figure 7, when the spectrum pricing of the primary base station 1 gradually increases in a certain range, because a high price can obtain more revenue, the income also increases; however, when the profit value exceeds a peak point, because the secondary network will choose a cheaper primary station to rent subcarriers, it resulting in a reduced subcarrier requirement for the primary base station 1, so the revenue of the primary base station 1 will be reduced. In addition, with the increase in the price of another primary base station 2, the demand of the secondary network for the base station 1 also increases, therefore, the primary base station 1 can set a higher price to get greater benefits.

#### 5.1.3. Optimal Response Function and Nash Equilibrium

**Figure 8:**
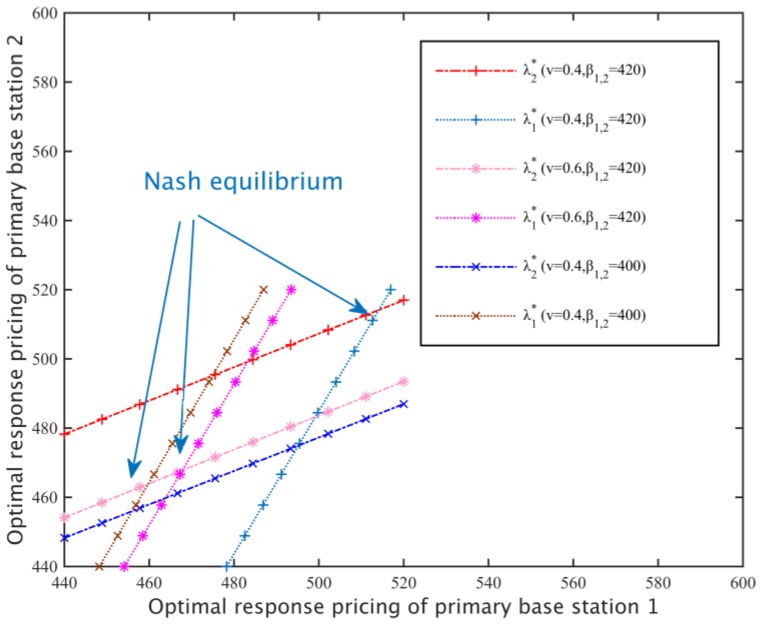
Optimal Response Function and Nash Equilibrium.

Setting different substitution rates *v*_1_ = 0.4, *v*_2_ = 0.6 and different primary base station loss coefficients *β*_1,2_ = 420, *β*_1,2_ = 400 (*β*_1,2_ represents *β*_1_ = *β*_2_), according to the optimal response function, that is $\lambda_{k}^{*}=\arg\max_{\lambda_{k}\in S_{k}}\;u_{k}^{PBS}(\lambda_{k},\lambda_{-k}^{*}),(\forall k\in M)$, Figure 8 can be drawn. The Nash equilibrium is located at the junction of the optimal response function of the different primary base stations, namely, the equation set (12). The less the loss coefficient of a primary base station, the smaller the rental per unit bandwidth caused by loss, and the primary base station is more willing to lower the price of spectrum resources, therefore, the Nash equilibrium price is lower. In addition, the substitution rate also affects the location of the primary base station pricing and the Nash equilibrium. The greater the replacement rate, the more difficult it is for the secondary network to switch from the spectrum of different primary base stations, and secondary base stations are more inclined to lower the price of rental spectrum resources, therefore, the price of the primary base station is lower when a Nash equilibrium exists.

#### 5.1.4. Dynamic Game

**Figure 9:**
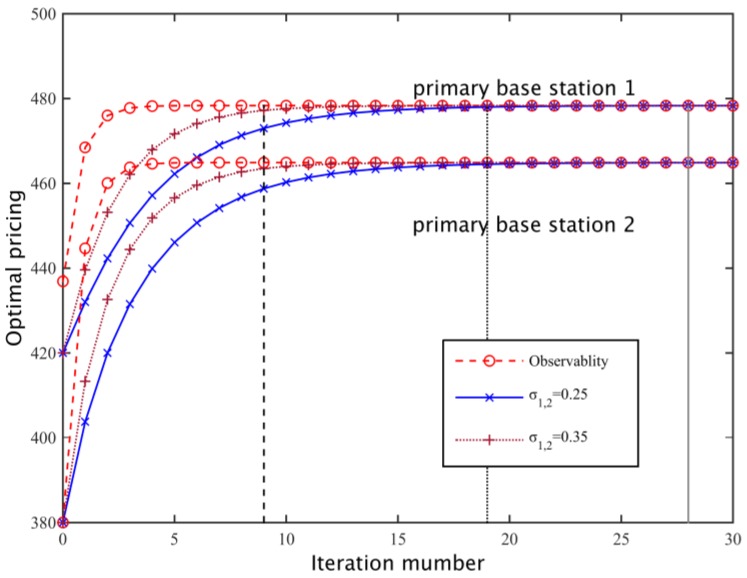
Dynamic game and Nash Equilibrium.

**Figure 10:**
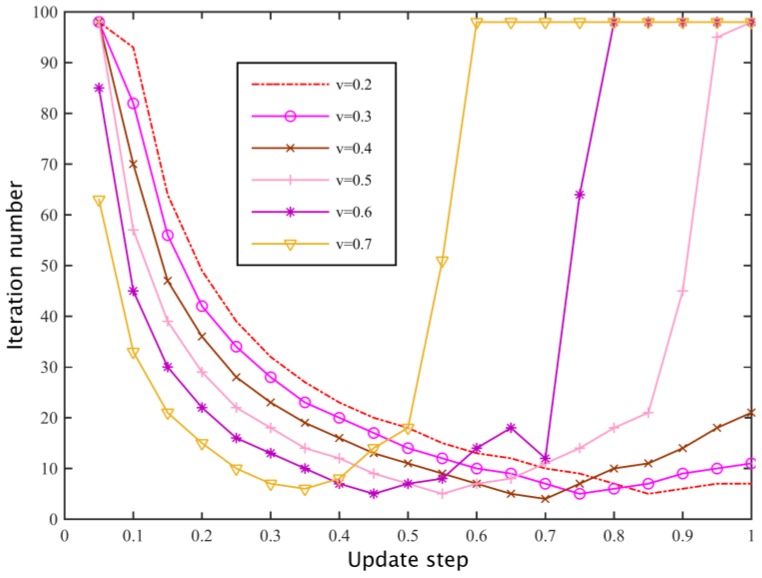
The relationship between the update step size and the number of iterations.

We set a secondary network spectrum substitution rate *v* = 0.4 and the loss coefficients of the two primary base stations are *β*_1_ = 420, *β*_2_ = 380. According to whether the primary base station can be observed in the history of the policy of other players, we study the process of the primary base station pricing through a dynamic game. For these two cases, the primary base station is respectively based on the optimal response strategy (Equation (13)) and learning strategy (Equation (14)) to dynamically update its spectrum pricing. Wherein, the sufficient conditions of a dynamic game for stability is $\sigma \in \left(0,\frac{\left(1-v\right)\left(2M-2\right)}{2M-1}\right)$, we select the learning strategy updating steps *σ*_1_ = *σ*_2_ = 0.25 and *σ*_1_ = *σ*_2_ = 0.35, we set the stability threshold *τ* = 0.01 and the initial pricing of the two primary base stations as *λ*_1_(0) = *β*_1_, *λ*_2_(0) = *β*_2_, then the dynamic game process is shown in Figure 9. As the primary base station is able to observe the historical policies of the other primary base station, a dynamic game can quickly converge to a Nash equilibrium based on an optimal response, but when a primary base station can only be based on the demand information fed back locally by secondary networks to update its pricing strategy, the rate of convergence of the dynamic game is largely dependent on the update step size, and as shown in Figure 10, when the replacement rate is lower, the dynamic game with larger update step has a faster convergence speed. When the replacement rate is higher, a smaller update step should be set to ensure the convergence rate. In addition, because of the high cost of the primary base loss coefficient of high rental per unit bandwidth, the optimal pricing of the equilibrium is higher than the primary base station of low loss coefficient.

#### 5.1.5. Loss Coefficient on Primary Base Station Revenue

**Figure 11:**
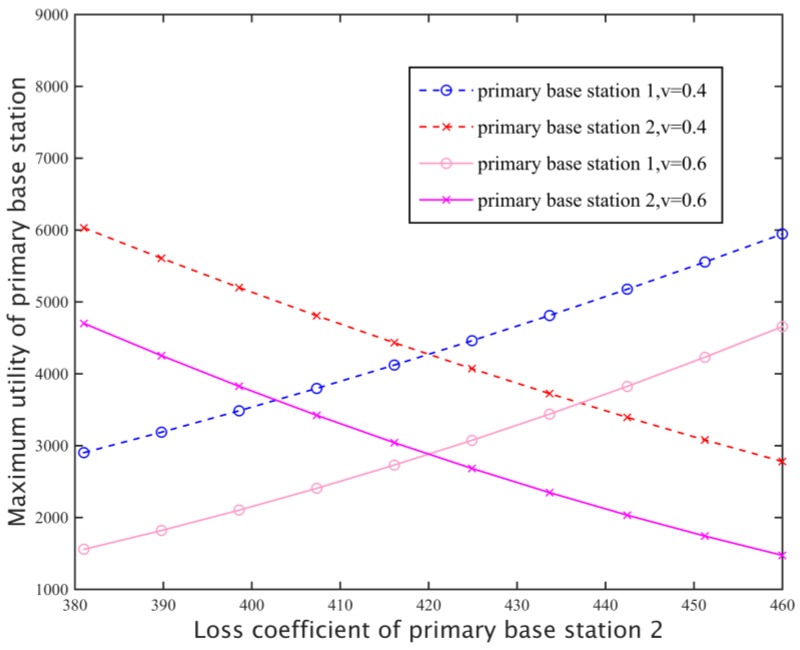
Maximum revenue of the primary base station.

Figure 11 shows the main effect of the loss coefficient of the primary base station 2 for its own and the revenue of the primary base station 1 for different replacement rates. When the loss coefficient of *β*_2_ gradually increases, the price of the primary base station 2 will be increased at a smaller rate than that of the *β*_2_, due to the fact the spectrum can be replaced between different base stations, so the corresponding spectrum rental also decreases, and the revenue of base stations is gradually reduced by *u_2_^PBS^*(***λ***) = (*λ*_2_ − *β*_2_)· D_2_(***λ***). On the other hand, the increase of *β*_2_ will increase the price of the base station 1, the rent amount is also increased, so the revenue increases. Not only that, but the replacement rate will also affect the primary base station revenue. For the same loss coefficient, increasing the replacement rate will decrease the optimal pricing of all primary base stations in the system, resulting in a decline in revenue.

#### 5.1.6. Spectrum Requirement Adjustment

**Figure 12:**
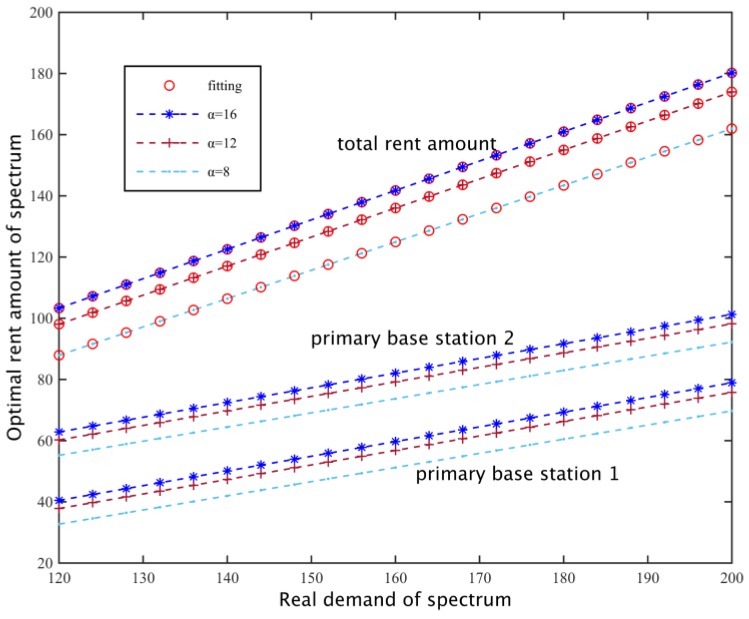
Secondary base station spectrum optimal rent number.

Setting the loss coefficient of two primary base stations as *β*_1_ = 420, *β*_2_ = 380 and replacement rate *v* = 0.4, we search for a particular preference factor, the relationship of the real needs of the subcarriers of secondary base stations and the final amount of the rental spectrum. As shown in Figure 12, when the spectrum need is the same, as the preference coefficient increases, the degree of preference of the secondary base station for the need is greater, so they tend to hire more subcarriers, thus increasing the spectrum demand of the secondary network, so the amount of rent subcarriers increases, and linear fitting for C with different requirements and its corresponding rental $q^{*}\left(C\right)$, so C and $q^{*}\left(C\right)$ show an approximately linear correlation. 

**Figure 13:**
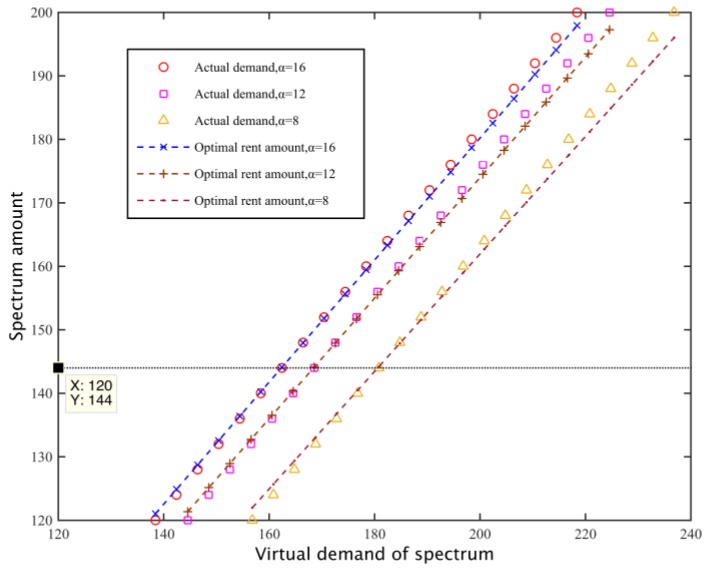
Spectrum demand and spectrum rent.

Subsequently, according to the adjustment strategy introduced in Section 3.5, we can determine the virtual demand for subcarrier $C^{\prime }$ and calculating the corresponding hire $q^{*}\left(C^{\prime }\right)$ based on $C^{\prime }$. Figure 13 describes the relationship between the virtual spectrum requirement $C^{\prime }$, the real spectrum needs C and the total amount of hiring spectrum $q^{*}\left(C^{\prime }\right)$ when C=144, $\varepsilon^{i}=0.5$. It can be seen that the adjustment of the virtual spectrum demand $C^{\prime }$ corresponds to the leased number of just $q^{*}\left(C^{\prime }\right)$ to meet the real needs of the network spectrum C.

### 5.2. Cooperation Spectrum Sharing

#### 5.2.1. The Degree of Satisfaction for the Secondary Network Communication Demand 

**Figure 14:**
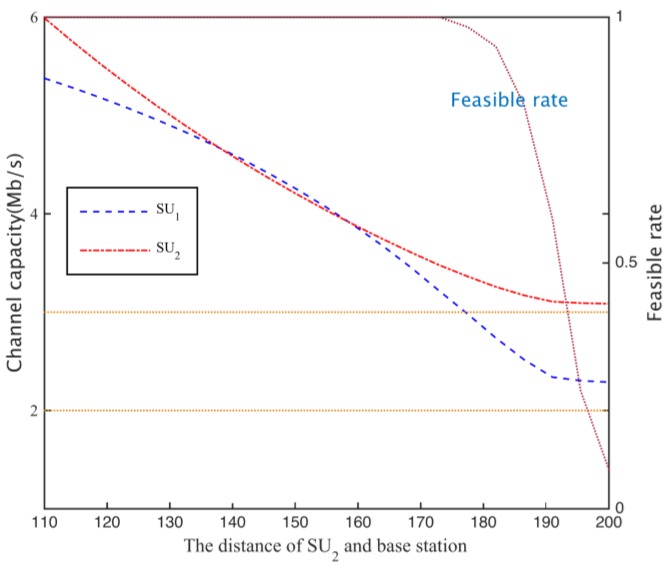
Each secondary user channel capacity.

When calculating the spectrum demand in the secondary network, if the secondary base station is based on the path loss of the secondary network boundary, we calculate the channel gain (case I). iIf the channel capacity requirement of the network is 5 Mb/s, the secondary base station needs to hire 144 subcarriers. Suppose secondary user $SU_{1}$ is located 100 m from the secondary base position, and the distance between $SU_{2}$ and secondary base stations changes between 110 m and 200 m, through a cooperative spectrum sharing scheme, each secondary the user can obtain the channel capacity as shown in Figure 14, where the horizontal dotted line in the graph is the secondary user channel capacity requirement, when the distance $D_{2}$ between the user $SU_{2}$ and the base station increases, the channel gain $h_{ij}{}^{2}$ and $D_{2}^{3}$ is a negative correlation, resulting in a decrease of $SU_{2}$ channel capacity. 

**Figure 15:**
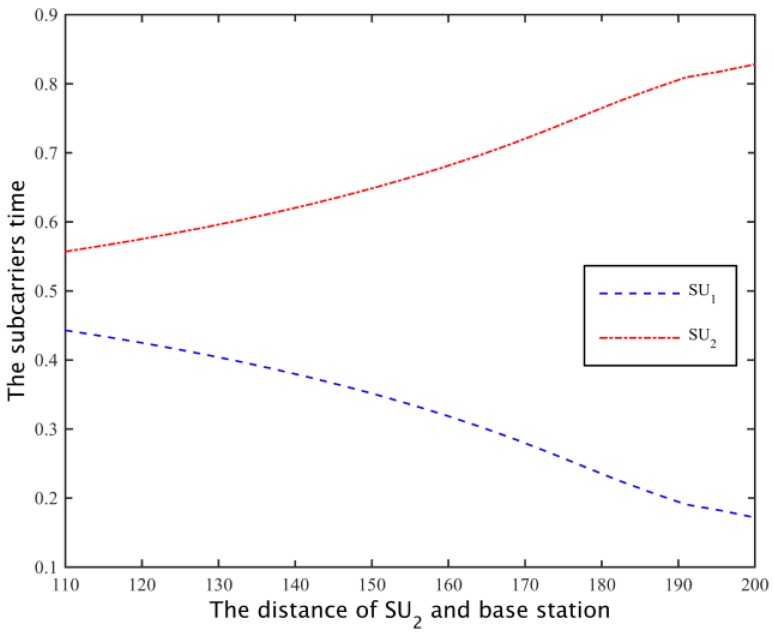
The optimal time assign of secondary users.

To obtain sufficient channel capacity for the secondary user $SU_{2}$, the spectrum sharing scheme can be used to reduce the time $SU_{1}$ is using subcarrier to increase the occupancy time of $SU_{2}$, so as to meet the channel capacity needs of $SU_{2}$, as shown in Figure 15. Thus, the cooperative spectrum sharing strategy based on the Nash bargaining scheme can allocate spectrum resources more equitably. In addition, when the distance of the SU_2_ distance is more than 20 m, the total channel capacity is higher than the demand, when $D_{2}>180\;m$, the path loss is increased, and the simulation channel can’t meet the increase of the number of $\sum_{i=1}^{N}\frac{R_{i}}{r_{i}^{*}}\leq 1$.

**Figure 16:**
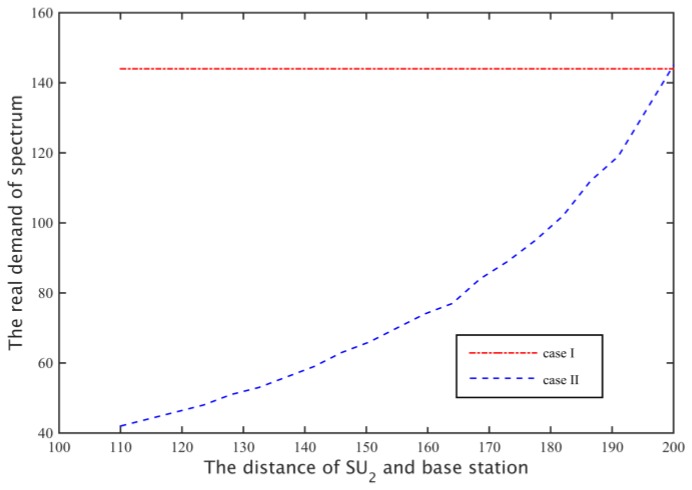
Spectrum requirements for secondary network of two cases.

When the secondary base station calculates the network spectrum demand, it considers the secondary user’s path loss of the secondary base station at the farthest distance (case II), so with the increase of the distance between the $SU_{2}$ and secondary base station, the spectrum leasing number that the channel capacity of 5 MB/s needs increases, as shown in Figure 16. 

**Figure 17:**
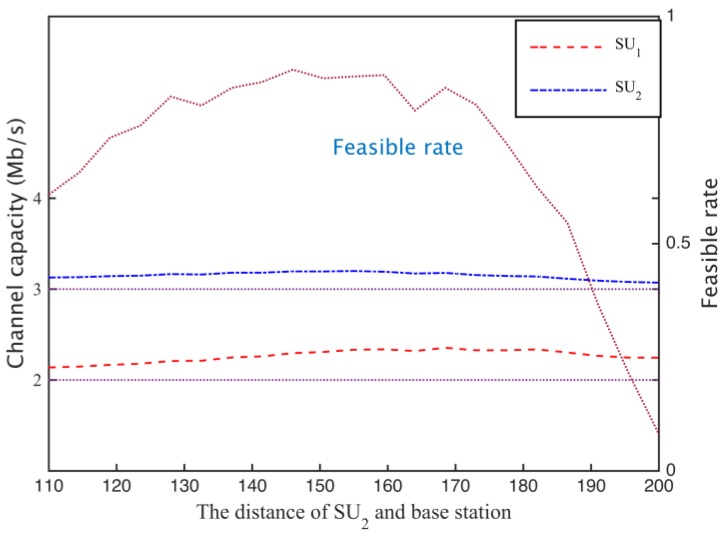
Each secondary user’s channel capacity.

**Figure 18:**
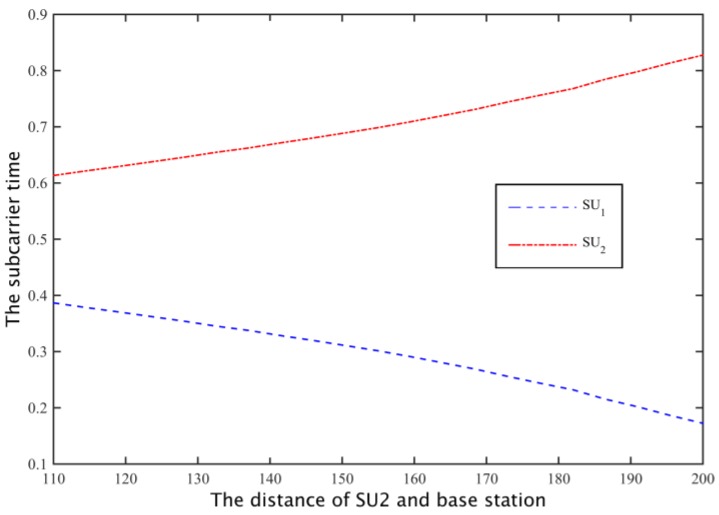
The optimal time assignments of secondary users.

**Figure 19:**
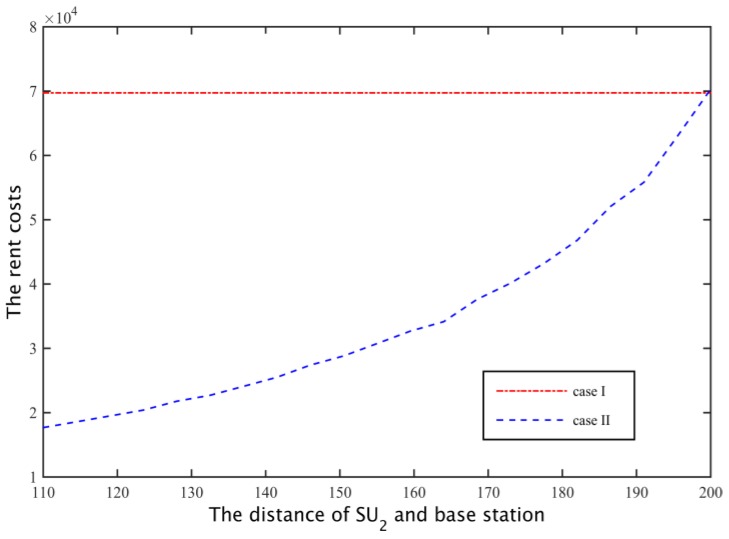
The cost of two secondary network leased spectrum cases.

Through cooperative spectrum sharing algorithms, two secondary users were able to get the channel capacity shown in Figure 17. As can be seen from the figure, $SU_{1}$ and $SU_{2}$ can get on average 13% and 5% higher demand for channel capacity, respectively, and with $SU_{2}$ away from the base station, the amount of spectrum that secondary base stations rent increases, to the transmission time that $SU_{1}$ uses for all the subcarriers to communicate is reduced, and the transmission time of $SU_{2}$ increases (Figure 18), but $SU_{2}$ is still able to maintain a higher demand for channel capacity. Moreover, from Figure 17 we can know that when the distance between the $SU_{2}$ and boundary is greater than 20 m, the probability of the secondary base station through the cooperative spectrum sharing algorithm is about 80%. Compared to the case I, the probability of the case II probability of the situation has declined, but the cost of the secondary base station is able to save 60%, as shown in Figure 19.

#### 5.2.2. The Fairness of the Cooperative Spectrum Sharing Scheme Based on NBS 

Assuming two secondary user’s spectrum needs are equal, both are 2.5 Mb/s, the distance of $SU_{1}$ and the secondary base station is 100 m, and the distance of $SU_{2}$ and the secondary base station is between 110 m and 200 m. Under the same constraint ((1) to (4) in Equation (25)), the cooperative spectrum sharing scheme is compared with the spectrum allocation scheme based on the maximum global rate [26] and the spectrum allocation scheme [27] based on the maximum degree of flatness. The objective function of the two kinds of spectrum sharing schemes are:

(39) $$Max\;rate:\max\sum_{i=1}^{N}r_{i}$$

(40) $$Max\;fairness:\max\min r_{i},i=1,2,L,N$$

where $r_{i}=\sum_{j\in SC_{i}}w\cdot \log_{2}(1+p_{ij}|h_{ij}{|}^{2}\cdot \eta )$.

**Figure 20:**
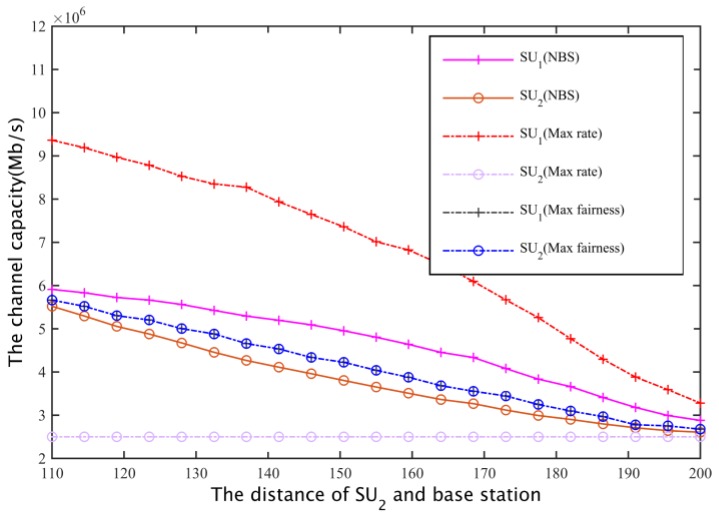
Secondary user channel capacity comparison I.

**Figure 21:**
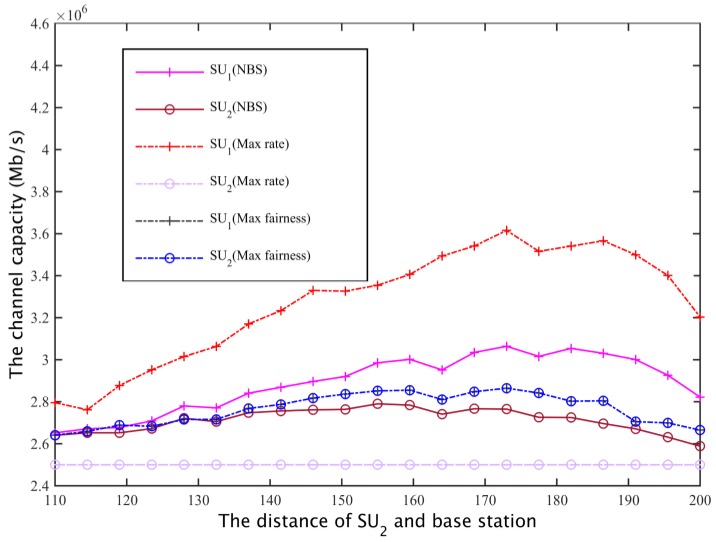
Secondary user channel capacity comparison II.

For two cases I and II (that is, a secondary base station based on the maximum coverage of the network, or based on the distance between the most remote users and the base station, to calculate the channel gain to determine the secondary network spectrum requirements), for these three spectrum allocation schemes, the secondary user $SU_{1}$, $SU_{2}$ channel capacity can be obtained as shown in Figure 20 and Figure 21. 

**Figure 22:**
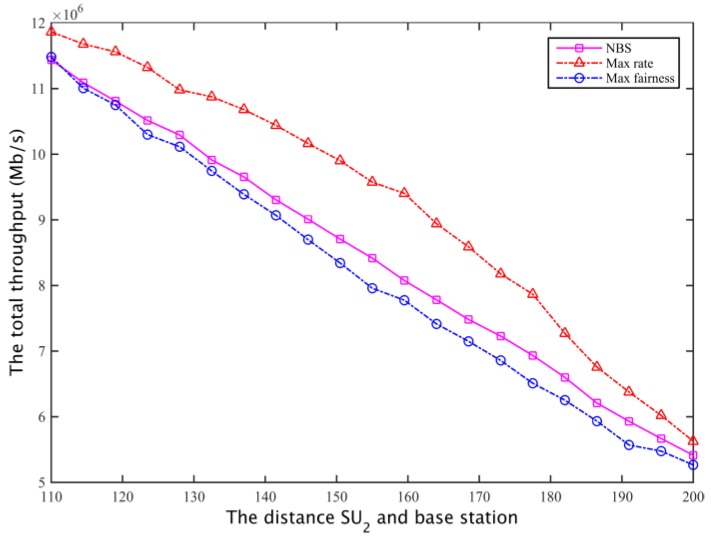
The comparison of total throughput for the secondary network I.

**Figure 23:**
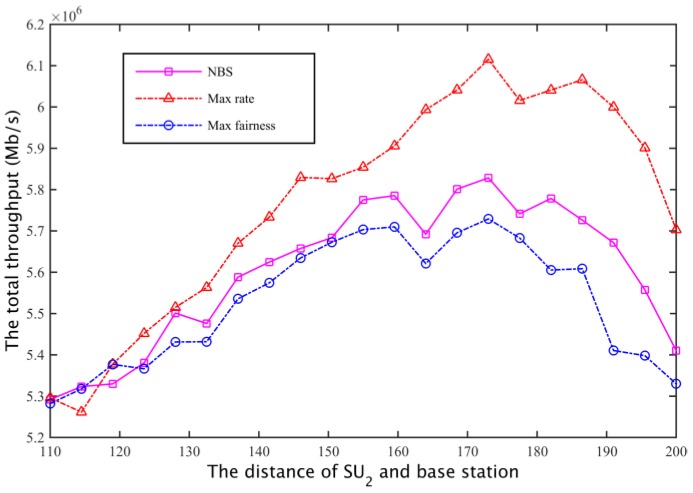
The comparison of total throughput for the secondary network II.

**Figure 24:**
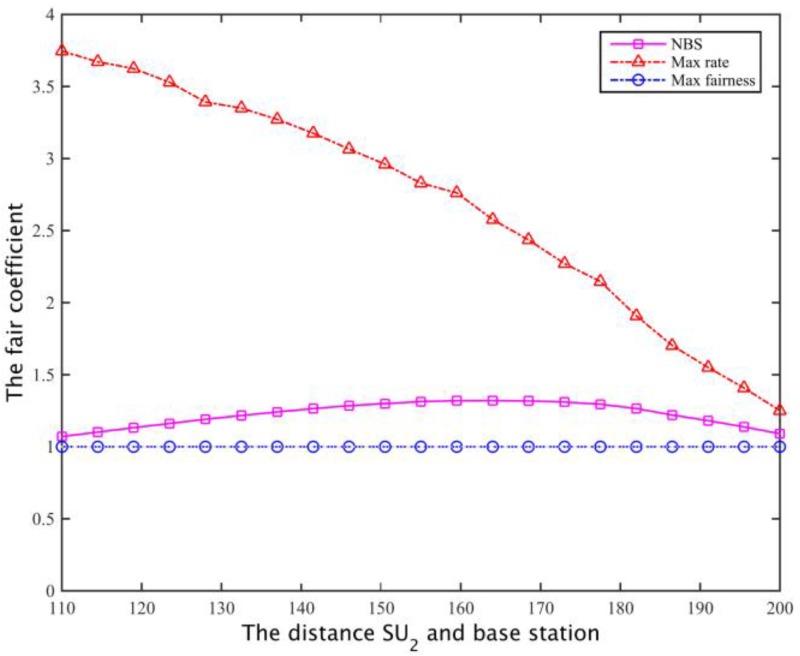
The comparison of fairness I.

**Figure 25:**
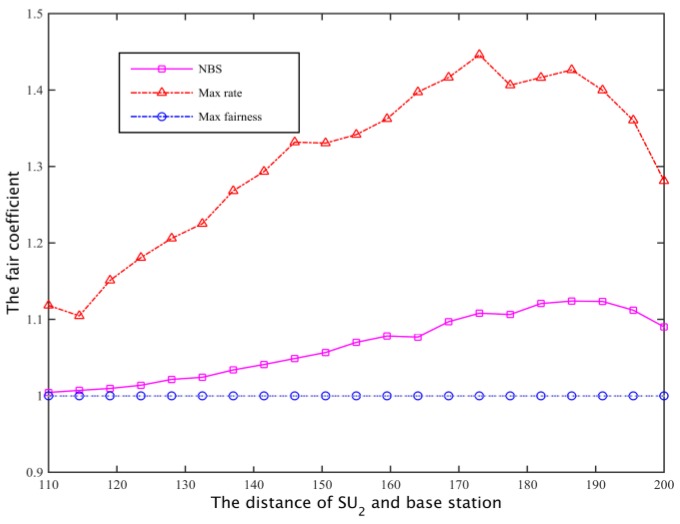
The comparison of fairness II.

The corresponding total throughput is compared with Figure 22 and Figure 23. Defining the fair coefficient is the ratio of the secondary user channel capacity, that is *r*_1_/*r*_2_, so the fairness of the three kinds of spectrum allocation scheme is obtained as shown in Figure 24 and Figure 25. 

In case I and case II, for the program based on the maximum global rate, the spectrum will allocated as much as possible to the secondary base station that is close to $SU_{1}$ while ensuring $SU_{2}$ just satisfy the constraints, that is, the secondary users whose channel gain is larger will obtain the maximum possible secondary network total throughput; the aim of the maximum fair spectrum allocation scheme is to reduce the total throughput of the secondary network, using the maximum fairness to ensure that the channel capacity obtained by $SU_{1}$ and $SU_{2}$ is the least. Relatively speaking, the NBS spectrum sharing scheme is able to compromise between the fair and spectrum efficiency, it can not only ensure the fair allocation of spectrum resources, but also enhance the spectrum utilization rate, finally obtaining a larger total network throughput.

## 6. Conclusions

With the popularity of the Internet of Things technology [28,29], the improvement of the communication efficiency and spectrum utilization of CRSNs has become a hot research topic, and dynamic spectrum sharing is the main countermeasure to improve spectrum utilization and alleviate the shortage of spectrum resources. However, due to the complexity of the application and the spectrum environment, most of current research on spectrum sharing is based on a large number of assumptions or lacks consideration of actual application scenarios. This paper presents a spectrum pricing and allocation model based on the Bertrand game theory. Spectrum pricing, subcarrier allocation, power control, and allocation issues are studied by synthesizing practical scenarios such as the frequency, power and time dimensions. It provides a practical and effective solution for spectrum sharing, and theory and experiments prove that the scheme can reflect the spectrum requirements of the secondary base stations and the benefit of the primary base station, and realize efficient spectrum sharing. It provides a better foundation and practical basis for the practical application of spectrum sharing, and provides theoretical support for the further development of CRSNs.

The simulation results also show that the model can reflect the demand of the spectrum of the secondary network. Primary base station spectrum pricing through a dynamic game of setting specific parameters, eventually converges to a Nash equilibrium; the adjusted spectrum requirements of secondary base station obtain the spectrum rent amount under market equilibrium, just to meet the needs of the network channel capacity, while in the secondary network, the secondary base stations are based on a cooperative spectrum sharing scheme, which can allocate spectrum resources fairly and efficiently.
